# Case Report: Percoronary device occlusion of right coronary artery fistula into left ventricle in an infant

**DOI:** 10.3389/fcvm.2025.1493724

**Published:** 2025-02-18

**Authors:** Shi-Bin Sun, Run-Tian Pai, Heng-Bao Wang, Zeeshan Farhaj, Yilei Xiao, Li Hongxin

**Affiliations:** ^1^ Department of Neurosurgery, Liaocheng People’s Hospital, Liaocheng, Shandong, China; ^2^ Department of Cardiovascular Surgery, Shandong Qianfoshan Hospital, Cheeloo College of Medicine, Shandong University, Jinan, Shandong, China; ^3^ Shandong Second Medical University, Weifang, Shandong, China

**Keywords:** coronary artery fistula, percoronary puncture, device occlusion, infant, transesophageal echocardiography guided

## Abstract

A 4-month-old male infant (weight 6 kg, height 67 cm) with heart failure was diagnosed with an isolated 8.9 mm right coronary artery fistula draining into the left ventricle (LV), identified via transthoracic echocardiography and computed tomography angiography. The large, tortuous, and aneurysmal fistula was treated using a minimally invasive percoronary approach, avoiding the high risks of surgery and the challenges of percutaneous closure. A 10 mm muscular ventricular septal occluder was deployed successfully. At 3 months, imaging showed reduced LV size, excellent device positioning, and complete fistula occlusion without thrombus formation. By 6 months, optimal remodeling was confirmed. Over 10 years of follow-up, the patient’s troponin I levels and electrocardiograms remained normal, with no ST-T abnormalities.

## Introduction

Large, tortuous, and complex coronary artery fistulas (CAFs) present significant challenges for both interventionists and surgeons (1–3). In a 4-month-old boy with an isolated, aneurysmal, and large CAF originating from the right coronary artery (RCA) and draining into the left ventricle (LV) (Supplementary Video S1), conventional surgery posed high risks, and a percutaneous intervention was difficult. This report details a minimally invasive percoronary device occlusion technique that offers a safe and effective solution for CAFs, regardless of their origin or drainage site.

## Case presentation

A 4-month-old male infant (weight 6 kg, height 67 cm) presented with heart failure and was diagnosed with an isolated CAF via transthoracic echocardiography and computed tomography angiography. The RCA was tortuous, aneurysmal, drained into the LV, and was without evident side branches. The drainage opening measured 8.9 mm and was located posterior to the heart, below the mitral valve annulus. A continuous murmur was auscultated at the left third to fourth intercostal space. Transthoracic echocardiography revealed a mean pressure gradient of 9 mmHg and LV dilation.

Due to the high morbidity of conventional open-heart surgery at this age and the challenges of percutaneous closure requiring a large arterial sheath, a percoronary approach was chosen.

**Figure 1:**
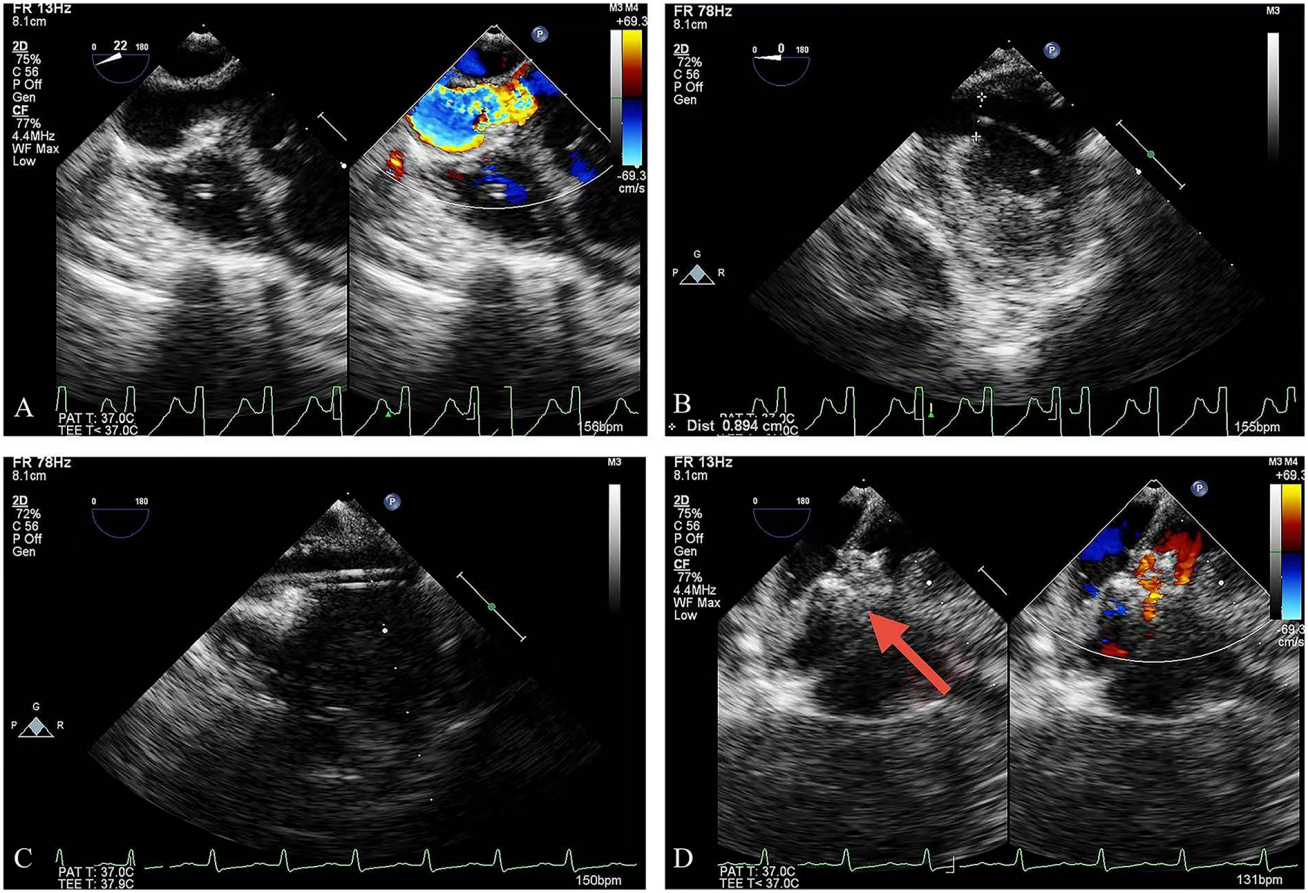
TEE guidance: **(A)** the outlet of the CAF; **(B)** the guidewire was advanced into the LV; **(C)** the delivery sheath was introduced over the guidewire; **(D)** deployment of the occlusion device (red arrow = device). TEE, transesophageal echocardiography; LV, left ventricle.

**Figure 2:**
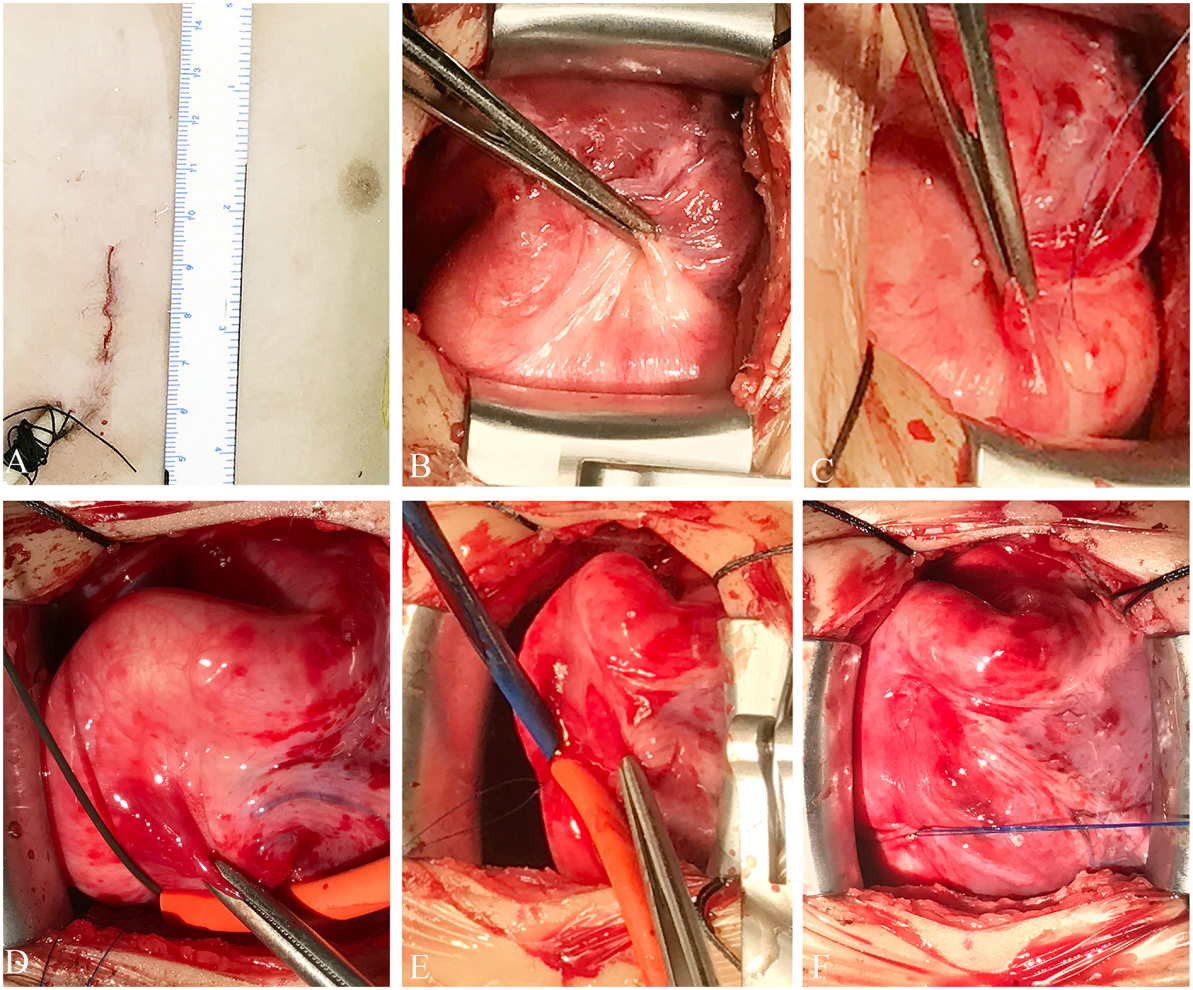
Schematic representation of the technique. **(A)** Incision site. **(B)** Surgical view of the fistula. **(C)** The purse-string suture is seen over the RCA. **(D)** Flexible guidewire introduced through the purse-string suture. **(E)** An 8-Fr short delivery sheath is introduced over the wire into the LV. **(F)** The purse-string suture is tied, showing complete hemostasis. LV, left ventricle; RCA, right coronary artery.

**Figure 3:**
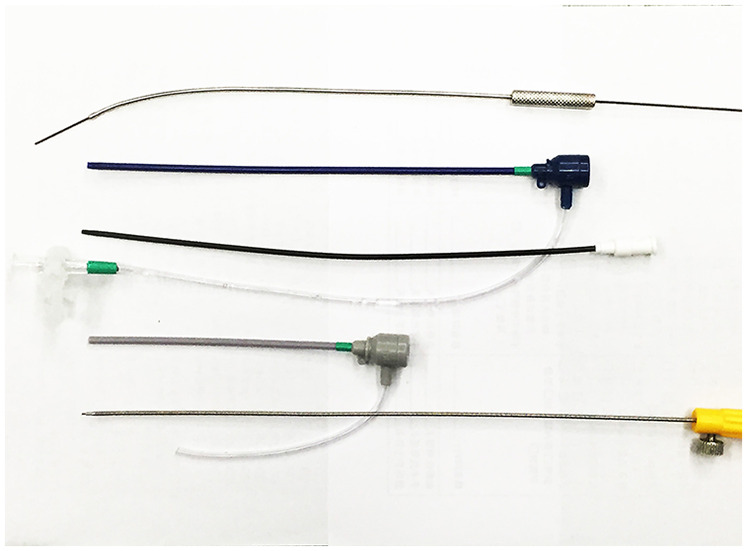
The delivery system comprising a hollow probe, short delivery sheath, loader sheath, and delivery cable.

The procedure was performed under general anesthesia with transesophageal echocardiographic (TEE) guidance (Figure 1). The patient was placed in a supine position, and a 3 cm lower mini-sternotomy incision was made. The pericardium was incised and suspended, and purse-string sutures were placed on the straight section of the RCA. The RCA was punctured and a flexible guidewire (16 cm; Lifetech, Shenzhen, China) was advanced through the RCA into the LV. An 8-Fr short delivery sheath was introduced into the LV over the guidewire (Figure 2). A 10 mm muscular ventricular septal occluder (retention disk diameter: 14 mm; Starway Medical Technology, Beijing) was deployed to occlude the CAF outlet. The delivery system used is shown here (Figure 3). A 15-min occlusion endurance test confirmed no ischemia, as indicated by continuous electrocardiogram monitoring. After confirming stability with a push-and-pull test, the occluder was released (Supplementary Video S2).

The patient stayed in the intensive care unit (ICU) overnight without ST-T segment abnormalities. Heparin was administered for 24 h postoperatively. The patient was discharged on postoperative day 7 and was prescribed aspirin (4 mg/kg/day).

**Figure 4:**
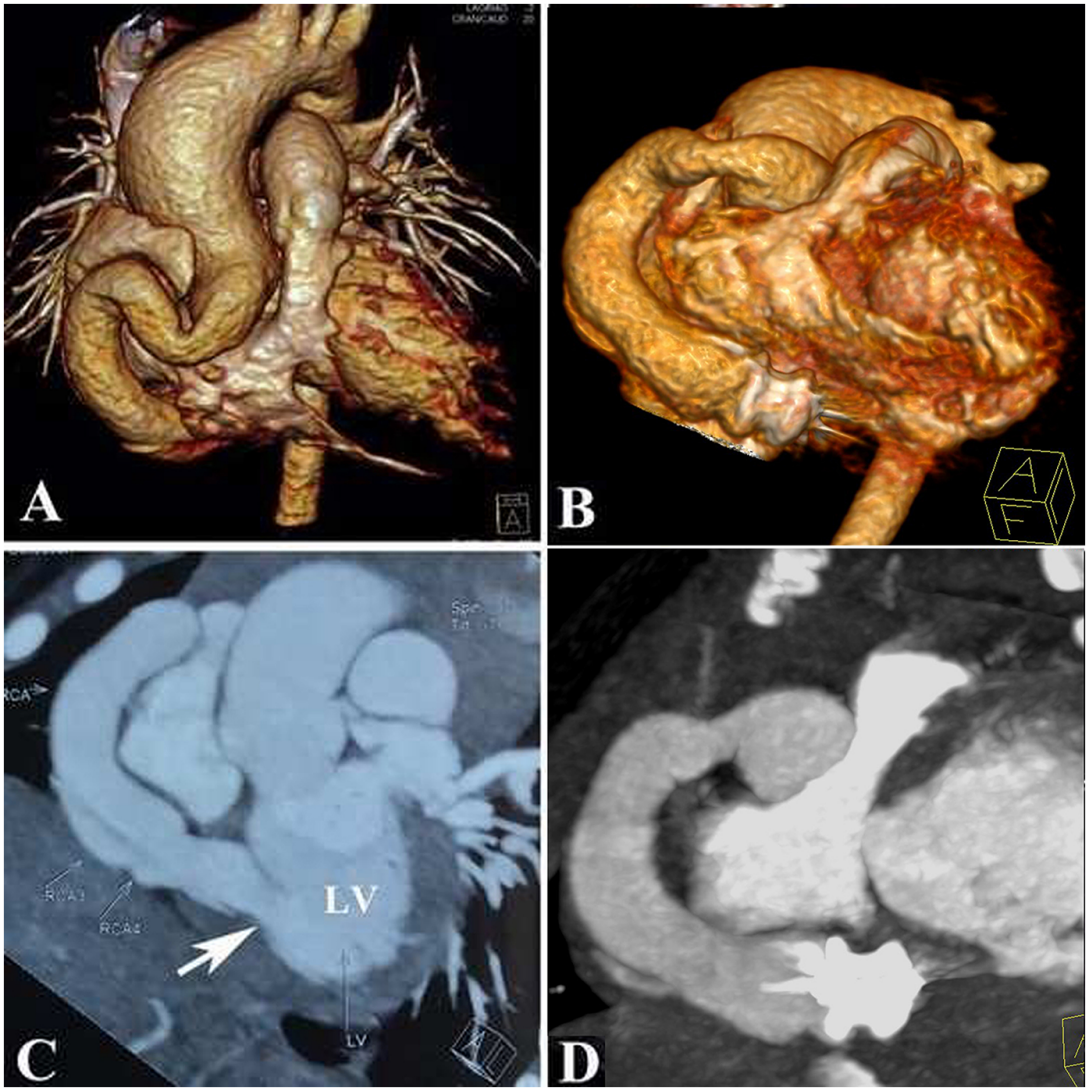
Anatomy of the coronary artery fistula showed by a computed tomographic angiography: **(A,C)**: Pre-procedure; **(B,D)**: Post-procedure.

At the 3-month follow-up, computed tomography angiography, and echocardiography showed decreased LV size, excellent device positioning, and complete CAF occlusion without thrombus formation (Figure 4). The RCA had no branches; therefore, we discontinued the aspirin after a departmental meeting. By 6 months, optimal remodeling was achieved, as evidenced by imaging studies (Supplementary Video S3). Troponin I levels and electrocardiograms remained normal during 10 years of follow-up, with no ST-T segment abnormalities observed. A short video demonstrates how the procedure is carried out (Supplementary Video S4).

## Discussion

Surgical intervention in this infant would have caused significant trauma, as it would have included sternotomy, cardiopulmonary bypass, and prolonged ICU stay, all of which are well-documented risks (2). In addition, the transcatheter approach was constrained by the challenges of arterial access and the inability to establish an arteriovenous circuit, as the fistula terminated in the LV. Creating such a circuit would have required advanced materials and large-profile devices, increasing the risk of electrical disturbances. The extensive use of fluoroscopy and contrast in such cases poses further risks, especially in infants.

Recent advances, including using microcoils and microcatheters, have shown promise even in small children (3). Combining these with our approach could provide a viable salvage strategy for failed transcatheter interventions or cases where surgery is the only alternative. Retrospective studies spanning three decades highlight that surgical closure is not commonly utilized, underscoring the need for less invasive options (3).

Treatment options for CAF include repair with cardiopulmonary bypass, ligation without cardiopulmonary bypass, and transcatheter closure using coiling, device occlusion, or covered stents. The choice depends on factors such as patient age, body weight, fistula size and tortuosity, termination site, coronary anatomy, and the number of fistulas (4, 5). Traditional surgery in small children is associated with significant morbidity, trauma, and complications (6).

In this case, the tortuous RCA draining into the LV, the large fistula opening, and the patient's low body weight made the transcatheter approach impractical. Open-heart surgery posed high morbidity risks, and percutaneous device closure was challenging due to the need for large arterial sheaths.

Previously, we reported CAF occlusion using right or left parasternal intercostal incisions, tailored to the fistula's origin, course, and termination (7, 8). This demonstrates the flexibility of our approach to ensure consistent outcomes while accommodating diverse patient anatomies. Our methods, which include peratrial, perventricular, and percoronary techniques, enable the occlusion of a wide range of CAFs, regardless of their origin, termination, or the patient's age and body weight.

CAF cases with specific characteristics, such as drainage into the right ventricle or other cavities and/or superficially dilated coronary segments (e.g., RCA or left anterior descending artery), may also benefit from this technique. Managing rare and complex CAFs requires expertise, but our approach simplifies the process without sacrificing efficacy.

In conclusion, the percoronary device occlusion of CAF offers the following benefits: no fluoroscopy or contrast agent use, no cardiopulmonary bypass, no age or weight limitations, and minimal technical requirements. This technique provides a safe, effective, and less invasive alternative therapy for selected patients.

## Data Availability

The raw data supporting the conclusions of this article will be made available by the authors, without undue reservation.

## Supplementary material

Supplementary Video S1 The CT scan before operation.

Supplementary Video S2 The procedure of device deployment with the TEE guidance.

Supplementary Video S3 The CT scan after operation showing optimal remodeling.

Supplementary Video S4 The video demonstrates the procedure done in Operation room, device is seen loading into the delivery sheath, followed by loading sheath removal while the assistant keeps the safety-suture stretched at side to avoid entanglement. The device is then deployed under the transesophageal echocardiography.
